# Influence of drying temperature on coconut-fibers

**DOI:** 10.1038/s41598-024-56596-z

**Published:** 2024-03-18

**Authors:** Flavia R. Bianchi Martinelli, Marcos Gomes Pariz, Rodolfo de Andrade, Saulo Rocha Ferreira, Francisco A. Marques, Sergio N. Monteiro, Afonso R. G. de Azevedo

**Affiliations:** 1 Civil Engineering and Buildings Department, IFES – Instituto Federal do Espírito Santo, Av. Vitória, 1729, Vitória, RJ 29040-780 Brazil; 2 IFES – Federal Institute of Espírito Santo, Av. Vitória, 1729, Vitória, RJ 29040-780 Brazil; 3 Civil Engineering Department, Federal University of Lavras, CP 3037, Lavras, MG 37200-900 Brazil; 4 Mechanics Department, IFES – Instituto Federal do Espírito Santo, Av. Vitória, 1729, Vitória, RJ 29040-780 Brazil; 5 Department of Materials Science, IME – Military Institute of Engineering, Square General Tibúrcio, 80, Rio de Janeiro, RJ 22290-270 Brazil; 6 LECIV – Civil Engineering Laboratory, UENF – State University of the North in Rio de Janeiro, Av. Alberto Lamego, 2000, Campos Dos Goytacazes, RJ 28013-602 Brazil

**Keywords:** Natural fibers, Drying, Coconut-fiber, Cementitious composites, Engineering, Materials science

## Abstract

The use of natural fibers in cementitious composites has been gaining prominence in engineering. The natural lignocellulosic fibers (NLFs) used in these composites have advantages such as reduced density, reduced fragmentation and concrete cracking, thus improving flexural performance and durability. Coconut-fiber is one of those natural fibers and its use presents technical, ecological, social and economic benefits, as it is improperly disposed of, representing a large waste of natural resources, in addition to causing environmental pollution.. Thus, composites reinforced with natural fibers are promising materials for the construction industry, as in addition to meeting the sustainability of buildings, there will also be a reduction in urban solid waste generated and gains for structures with the use of environmentally friendly materials that meet to active efforts and with greater durability. This work aims to evaluate the tensile behavior of green coconut-fibers subjected to different drying temperatures through chemical, thermal (TG/DSC), morphological, visual and mechanical analysis. Drying temperatures of 70 °C, 100 °C and 130 °C were analyzed and the results indicated that the drying temperature at 70 °C was satisfactory, providing fiber-reinforced composites with good tensile strength, combined with good ductility.

## Introduction

In Brazil, the total generation of urban solid waste in 2021 was approximately 82 million tons, equivalent to 390 kg/person/year^1^. Of the urban solid waste generated, attention should be paid to the green coconut, a tropical fruit whose production record in Brazil was approximately 2.65 million tons in 2016^2^. It is recorded that 70% of the garbage collected on Brazilian beaches is composed of coconut husks, and that factors such as proliferation of vectors and long period for decomposition contribute to the reduction of the useful life of landfills in which coconut husks are discarded^3,4^

Although Brazil is a large consumer of coconuts, only part of it is used for food^2^. The pulp of the green coconut is edible, but consumption of the fruit *in natura* is often limited to water. This results in the disposal of thousands of tons a month, representing a huge waste of natural resources. Thus, the use of green coconut residue, especially its husk fibers, has technical, ecological, social and economic benefits.

Natural fibers are ecological and low-cost materials, considered new, effective, alternative materials and their field of use is quite wide, from applications in tapestry, ornamental pieces to the textile industry. Specifically in civil construction, natural fibers are used in cement matrices in the production of roofing and cladding elements, particle board sheets, green roofs, reinforcement and rehabilitation of masonry walls and housing components^5^.

As for the addition of 0.5% by volume of green coconut-fibers in cementitious matrices, there was an increase in compressive strength of around 15% and tensile strength of 20%, when compared to conventional concrete^3^. On the other hand, considering the compressive strength with the addition of 4% by volume of coconut-fiber to the concrete, this resistance was reduced by around 50%, while in the flexural resistance there was an increase of 50%^6–14^. In addition to dispersed fiber, there are studies to reinforce concrete walls using ropes composed of coconut-fibers in order to increase the stability of structures against earthquakes^15^. Other studies used coir-fibers in concrete to improve its properties under impact load, reducing concrete spalling and cracking^16^ Rumbayan et al.^17^ found that 0.25% coconut-fiber provided an improvement of approximately 19% in compressive and flexural strength in 28 days. It was found in this study that the greater the amount of coconut-fiber in the concrete, the lower the tensile strength and that with the presence of fibers in the concrete there is lower workability.

Various natural fibers are used in cementitious composites such as oil palm broom fibers, açaí, bamboo, curauá, etc. Oil palm broom fibers when subjected to treatments show improvements in their performance, for example using treatment with an alkaline concentration of 6% NaOH at room temperature for 48 hours showed improvements in tensile strength and in elastic modulus of up to 60% and 65%, respectively. Already considering silane treatment, Momoh et al.^18^ found that the ideal recommendation is 3% silane for 24 h at room temperature, which resulted in up to 60% improvement in tensile strength and a reduction of 11 % in water absorption. Hot water treatment for 30 min at 100 °C resulted in a 40% increase in tensile strength. Açaí fiber has a tensile strength of 17.8 MPa whereas curauá fiber already has a tensile strength of 488 to 752 MPa.

As most natural lignocellulosic fibers (NLFs), the coconut-fibers are highly hygroscopic and their use, when wet, strongly affects the properties of these materials^17^. As its mechanical properties are dependent on the moisture content and considering its use as reinforcement in composites, this moisture content must be reduced through the drying process. In general, artificial drying with ovens or dryers is used^19^. Therefore, the objective of this study is to analyze the tensile behavior of green coconut-fibers subjected to different drying temperatures (70 °C, 100 °C and 130 °C) in comparison with natural fibers (without drying process). It is therefore expected that it will be possible to define the most suitable drying temperature for the application of green coconut-fibers in cementitious composites.

## Coconut-fibre-reinforced cementitious composites

Coconut-fiber is an NLF obtained from the fibrous mesocarp of the coconut, the fruit of the coconut tree (*Cocos nucifera*). The chemical composition of coconut-fiber consists of: 36 to 43% by weight of cellulose, 41 to 45% by weight of lignin and 0.15 to 0.25% by weight of hemicellulose^20^. Cellulose is the main chemical constituent responsible for the stability and strength of fibers^21–23^. Coconut-fiber is used as an engineering material due to its high tensile strength and low modulus of elasticity compared to other fibers^20^.

In coconut-fiber there is a smaller amount of cellulose, but the lignin content is higher, unlike most NLFs. Lignin provides compressive strength to cellular tissue and fibers, stiffening the cell wall and protecting against physical and chemical damage. Lignin has the function of uniting plant tissues, reinforcing the fiber's cell wall, protecting it from physical and chemical damage^23^.

Concrete elements reinforced with coconut-fibers have greater resistance to fracture due to this greater flexibility. In addition, it has greater durability because when the concrete cracks, it does not flake^24^. Compared to other natural fibers, coconut-fiber can deform 4 to 6 times more, resulting in a reduction in cracking, meaning that concrete with coconut-fibers, compared to traditional concrete, has better flexural behavior and impact, but a reduction in compressive strength and workability may occur.

Cellulosic fibers have a hydrophilic nature (affinity for water) under natural conditions. The moisture content in the fibers can negatively influence the mechanical behavior of natural fiber composites. One way to improve this mechanical behavior is through surface modification of the fiber by physical or chemical methods^25–27^.

According to Gholampour and Ozbakkaloglu^22^, due to the high sensitivity of natural fibers to humidity, moisture absorption results in delamination between the matrix and the fiber, reducing the mechanical properties of the composite. This is attributed to the fact that due to the presence of non-cellulosic components (i.e. pectin, lignin and hemicelluloses), natural fibers in nature are polar and hydrophilic and therefore create active conditions (i.e. hydroxyl accessibility, OH, and carboxylic acid groups) for water absorption. According to Tian et al.^5^ in alkaline cementitious environment, the lignin of natural fibers is decomposed, causing significant degradation in the strength of concrete reinforced with natural fiber. Natural fibers can only retain about 20% of their original tensile strength after being attacked by NaOH or Ca(OH)_2_ solutions.

The natural moisture content of NLFs ranges from 10 to 18%. The moisture in the fibers occurs because they are plant cells in association. Therefore, drying these materials in ovens must be carried out, but at temperatures above 100 °C, there is a risk of degradation^28,29^.

Voids are also part of the structure of NLFs. The greater the degree of porosity, the greater the moisture absorption by the fiber. The humidity of the fibers influences the tensile strength, the degree of crystallinity and swelling, which facilitates their degradation in alkaline environments^9^.

## Fiber drying

NLFs, when extracted from the plant, are wet and to reduce this moisture content they are subjected to drying. This drying process must be controlled as it is of great importance to guarantee the quality of the fibers, particularly in relation to mechanical resistance and coloring. Considering its use as reinforcement in composites, this moisture content must be reduced through a drying process (generally artificial drying with ovens or dryers), as the mechanical properties are dependent on the moisture content^17^.

During drying distinct waters are removed: (1) free (adsorbed to the material); (2) constitution (bound to proteins and carbohydrates); and (3) adsorbed on the surface of the colloidal particles. This removal of water helps to concentrate the chemical constituents. During drying, the external volume of materials is reduced, which is one of the most important physical changes^24,30^. There is also damage to the cellular structure of the materials, such as changes in shape and reduction in size, as there is loss of water in this drying process^30,31^.

The drying process involves diffusion of molecules and a flow of matter from a region of high concentration to a region of low concentration. Diniz et al.^19^ found that the drying of sisal fibers at lower temperatures was more controlled, guaranteeing a quality product, without strong changes in its pigmentation. For the drying temperature of 90 °C, a significant change in the color of the sisal fibers was observed at the end of the drying process.

For the green coconut mesocarp, from which the fibers are removed, with the increase in the drying temperature, there was a decrease in the specific volumes of micro and mesopores^24^. In addition, it was verified that in the interconnected type pores (bottleneck and cylindrical) during the drying process, the shape of these pores can hinder the migration of water to the surface, causing the water to remain partially adsorbed, leading to a decrease in the specific volumes of micro and mesopores. In micropores, water movement can be impeded, due to the formation of very strong capillary forces during the drying process. In the mesopores (capillary pores) water percolation can occur through the network that they form.

The drying process uses heat to remove water and volatiles present in the fibers by evaporation. Knowledge about drying is essential as fibers change their behavior from plastic to brittle in relation to the presence of moisture and volatiles. The chemical composition of the fiber also changes. During drying, there is an energetic effort to break the physical barriers of fiber conformation to remove moisture and volatiles, requiring the moisture to cross the fibrous mass through winding paths to the surface. This requires energy for displacement while also requiring latent heat for vaporization^10^.

The time required for the total removal of water from the coconut-fiber at 100 °C is directly proportional to its thickness and the shrinkage of the fibers after drying varies depending on the orientation of the fiber and its thickness. Shrinkage being greater for the widths and thicknesses where the fibers are parallel to each other^31^. This is probably because greater fiber packing is possible. The samples with a thickness equal to 5 mm remaining in an oven for 120 min at a temperature of 100 °C, showed a mass loss of 89.96%. This period being sufficient for no further mass variation. The 10 mm samples remained for 240 min with a mass loss of 89.95% and the samples with thicknesses equal to 15 mm remained for 360 minutes with a mass loss of 89.97%.

Still according to Fernandes et al.^32^, the mass loss variation was 0.02% between the studied samples, revealing that possible errors related to experimental manipulation were minimized. The total drying of the samples is independent of the area or volume, depending only on the time spent in the oven. Thus, the authors Fernandes et al.^32^ concluded that the time spent in the oven increases proportionally with the thickness of the sample, indicating that a greater amount of energy is required for drying the fibers and also found that the retraction of the fibers after drying decreased with the increase in the thickness of the fiber.

This work aims for the first time to analyze the tensile behavior of green coconut-fibers subjected to different drying temperatures (70 °C, 100 °C and 130 °C) in comparison with *in natura* fibers (without drying process). It is expected, therefore, be possible to define the most suitable drying temperature for the application of green coconut-fibers in cementitious composites.

## Materials and methods

The green coconut husks were obtained from vendors, that commercialized the coconut water, located at Praia de Camburi in Vitória, Brazil. The coconut husks were removed and the fibers extracted from the mesocarp, being separated and shredded manually. The fibers were not subjected to any chemical or physical treatment, they were only dried in a conventional oven at temperatures of 70 °C, 100 °C and 130 °C until mass constancy, that is, until two consecutive weighings spaced 1 h apart did not show mass variation greater than 0.1%. The total drying time was 180 minutes for fibers dried at 70 °C, 150 minutes for fibers dried at 100 °C and 100 min for fibers dried at 130 °C.

### Chemical analysis

The chemical analysis was carried out at the Plant Physiology Laboratory at the Federal University of Espírito Santo, Brazil, and the coconut-fibers were previously crushed in a knife mill, it is not necessary to specify the size of the particles.

Cellulose determination of the samples was performed according to Brendel et al.^33^ The methodology used for the determination of hemicellulose was recommended by Schädel et al.^34^. The determination of lignin was performed according to Dos Santos et al.^35^.

### Scanning electron microscopy

Morphological analysis was performed by SEM with EDS. To obtain the micrographs, a JEOL scanning electron microscope, model JSM6610LV was used with an acceleration voltage of 20 kV and magnification of 150 × and 500 ×. The analysis was carried out for fibers *in natura* (without drying process) and for fibers dried in an oven at 70 °C, 100 °C and 130 °C. All samples were metallized with a gold coating.

### Optical microscopy

The visual analysis and the determination of the average diameter of the green coconut-fibers were carried out in an optical microscope, Leica, Model EZ4 HD. The images were obtained at a magnification of 25 ×, and the recording was performed using the Leica LAS EZ 3.4 DVD 272 software.

In order to determine the average diameter of the fibers, measurements were taken at 3 different positions along their length, in addition to measurements close to the breaking point after the direct traction test. At measured points, a rotation of 90° around the fiber longitudinal axis was performed and the recording was repeated. These measurements were made for the two parts of the fiber after rupture in the direct tensile test.

**Figure 1:**
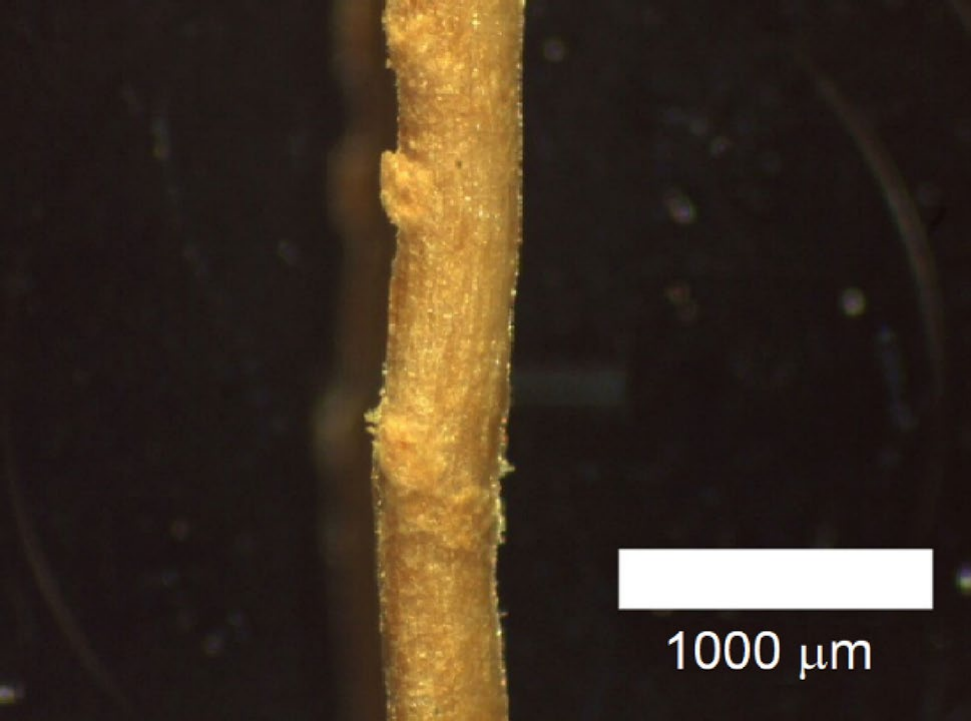
Micrograph of coconut-fiber with the graduated ruler.

To carry out each measurement of the diameter in the optical microscope, a graduated ruler was used, provided by the manufacturer (Fig. 1) which identifies the scale of 1000 μm. The registered measure was obtained through correlation between the graduated ruler and the width registered in each image.

The diameter used in this work was the average of the recorded measurements. And considering the cross section of the circular fiber, the average area for each tested fiber was determined.

### Thermogravimetric analysis and differential scanning calorimetry (TG-DSC)

The TGA was performed using a thermal analyzer, model 449 F3 Jupiter from Netzsch, performs simultaneous thermal analysis of materials: thermogravimetric (TG) and DSC. Thermogravimetry analysis (TGA) and differential scanning calorimetry (DSC) were used to analyze the thermal behavior of coconut-fiber previously subjected to drying at different temperatures. With a heating rate of 10 °C/min in a dynamic argon atmosphere, with a flow rate of 60 ml/min in the oven and 20 ml/min in the safety system, in the temperature range of 20 to 900 °C. A sample mass of 10 ± 1 mg in alumina non was used in each test. The samples were tested *in natura* and dried at 70 °C, 100 °C and 130 °C, previously crushed in a knife mill.

#### Tensile tests

**Figure 2:**
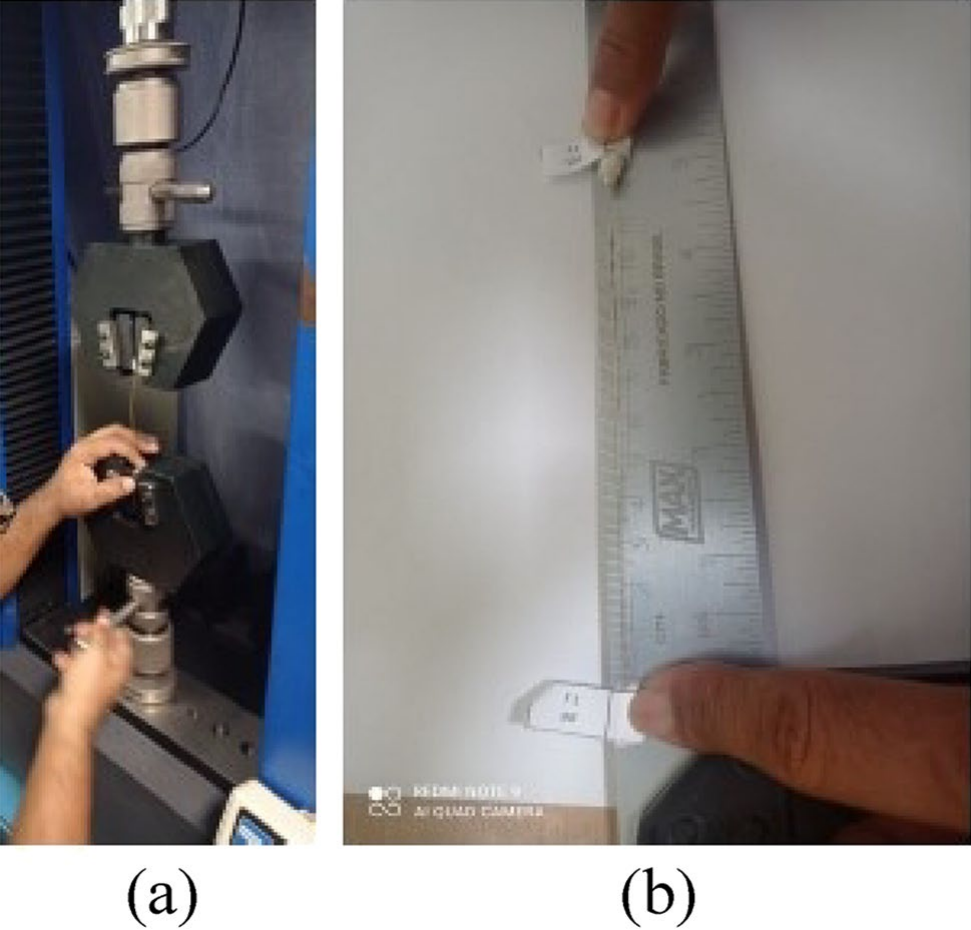
Tensile test of coconut-fibers: (**a**) Universal Emic machine and (**b**) Fibers identified for testing.

The tensile tests on the fibers were carried out at RT in a universal machine Emic DL-10.000, servo-controlled, load cell TRD 22 with capacity equal to 100 kgf (Fig. 2a).

A selection of 10 fibers *in natura*, 10 fibers dried in ovens at 100 °C, 9 fibers dried in ovens at 130 °C and 9 fibers dried in ovens at 70 °C were tested, with a free length between grips of 12 cm (Fig. 2b), with displacement control of the test machine beam at a rate of 0.5 mm/min. In order to avoid a possible rupture due to stress concentration caused by the pressure of the machine's jaws, an adhesive tape was glued to the ends of the fibers.

Besides, analysis of variance (ANOVA) was performed with the Minitab 19.1 statistical analysis program, considering a completely randomized design (DIC). The purpose was to verify the existence of statistical significance in the tests carried out considering the drying temperatures. Once the statistical difference was confirmed, Tukey's mean comparison test (*p* ≤ 0.05) was performed to verify which drying temperature obtained the best results for the tensile strength of the fibers.

## Results and discussions

### Chemical analysis

The average experimental values of cellulose, hemicellulose and lignin for dry fibers are listed in Table 1. By the methods employed, it was not possible to test the fibers *in natura* due to the high moisture content present in these fibers, however, Table 2 shows the chemical composition of the *in natura* fiber researched by several authors.

**Table 1:** Mean values of chemical analysis of dried coconut-fibers.

Drying temperature (°C)	Cellulose (% weight)	Hemicellulose (% weight)	Lignin (% weight)
70	14.39 ± 1.18	3.59 ± 1.92	8.57 ± 2.27
100	15.50 ± 1.14	3.24 ± 0.84	11.44 ± 1.30
130	18.47 ± 1.71	1.15 ± 1.32	13.23 ± 1.05

**Table 2:** Chemical composition of *in natura* coconut-fiber.

Authors	Cellulose (% weight)	Hemicellulose (% weight)	Lignin (% weight)
Aragão et al.^36^	23–43	3–12	35–45
Ramamoorthy et al.^37^	36–43	0.15–0.25	41–45
Malkapuram et al.^38^	32–43	0.15–0.25	40–45
Marafon et al.^39^	35.52	33.41	22.25
John and Anandjiwala^40^	33.2–43	31.1	20.5
Ramakrishna and Sundararajan^41^	33.2	31.1	20.5
Ars_ene et al.^42^	21.46	12.36	46.48
Kochova et al.^43^	36.6	37	22.2
Fuqua et al.^44^	32–47	0.3–20	31–45

The main constituents of lignocellulosic materials decompose between the following temperatures: hemicelluloses: 200 and 260 °C; cellulose: 240 and 360 °C; lignin: 280 and 360 °C^45^. Thus, the temperatures employed in this study for drying the fibers did not promote the degradation of these components.

Depending on the cultivation conditions, location or age of the fruit, it can cause changes in the chemical composition of lignocellulosic plant fibers^12^. The lignin content in coconut-fibers is 20% in the young fruit and 35% in the mature fruit^44^. The variability of this chemical composition of *in natura* coconut-fiber can be seen in Table 2.

The variability of the experimental results found (Table 1) corroborates the literature that indicates the dispersion of cellulose, hemicellulose and lignin values due to the nature of the vegetable fibers (Table 2). The temperatures employed being lower than the degradation temperatures of these constituent materials, it does not justify the differences found.

These changes in the chemical composition of the fibers were due to their drying process, as according to Fernandes et al.^32^ the chemical composition of the fiber also changes with drying. During drying, there is an energetic effort to break the physical barriers of fiber conformation to remove moisture and volatiles, requiring the moisture to cross the fibrous mass through winding paths to the surface.

In studies of fiber-matrix interfacial behavior, Tian et al.^5^ and Ramli et al.^46^ verified that the natural fiber, during the mixing process with the matrix, partially absorbs free water, causing it to swell. When the fiber shrinks, there will be a gap in the fiber/matrix interfacial transition zone, and when the gap becomes larger, the friction force decreases.

The interfacial connection between the fiber and the concrete matrix is considered a factor that affects the mechanical properties of the composite, consequently affecting its durability, with the main failure mechanism being the pulling out of the fibers. Interfacial bonding in cement-based fiber composites can be affected by several variables such as water/cement ratio, porosity, fiber shape, morphology and compaction^5,46^.

According to Methacanon et al.^29^ and John and Thomas^47^, the moisture absorption of natural fibers is related to the hemicellulose content of the fiber, and the higher this content, the greater the moisture absorption.

Several studies have been carried out to improve the mechanical properties of natural fiber and, consequently, the physical and mechanical properties of cementitious composites reinforced with natural fiber, subjecting the fibers to physical (hornification, corona, cold plasma, ultraviolet rays, heat treatments with electron radiation, among others) or chemical (alkaline treatment or mercerization, acetylation, silane, permanganate, impregnation, among others) treatments. Physical treatments on fibers modify their structural and surface properties without transforming their chemical composition, increasing mechanical adhesion between the natural fiber and the matrix, improving the interface without changing the chemical properties of the fibers. Chemical treatments of natural fibers reduce the inherent hydrophilic behavior of the fibers and improve the adhesion properties of the matrix and fiber. These treatments were not the subject of analysis in this study^22,25,26^.

### Scanning electron microscopy

**Figure 3:**
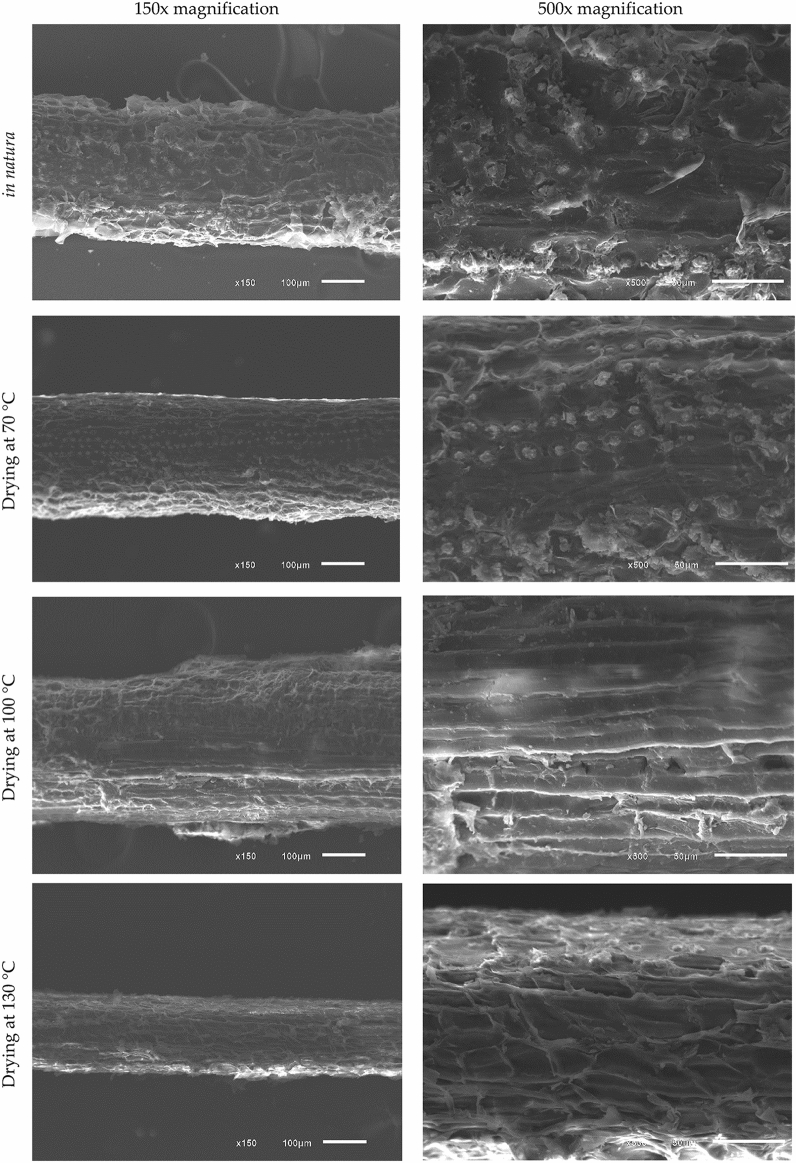
Micrographs of coconut-fibers at different drying temperatures: (**a**) 150 × magnification and (**b**) 500 × magnification.

Through the micrographs obtained by SEM for fresh coconut-fiber, Fig. 3, it can be seen that the fiber has a cylindrical shape and has surface roughness, which helps with resistance at the interface between the matrix and the fiber.

For coconut-fibers dried in an oven at 70 °C, 100 °C and 130 °C, it can be seen that the fiber has undergone surface changes, reducing the diameter with increasing temperature.

The study by Hwang et al.^14^ showed the rough surface and porous structure of coir-fiber, furthermore, indicating that the pretreatment process induced morphological changes that increased the void volume and surface roughness.

According to Kochova et al.^43^ pretreated fibers have a better interface with cement than untreated fiber, with less slip softening and more slip hardening behavior during the pull-out tests. Washed fibers also have a better interface than unwashed fibers due to the removal of unwanted constituents, wax and other impurities on the surface can affect the cement/fiber interaction^43^.

When an alkaline treatment is applied, it impacts the morphology, molecular and supramolecular properties of cellulose. Consequently, the fibers become more rigid, accessible and present changes in crystallinity and pore structure.

### Optical microscopy

The average diameters found for the coconut-fibers tested were:

− 410 ± 118 μm for *in natura* fibers;
− 325 ± 191 μm for fibers dried at 70 °C;
− 265 ± 60 μm for fibers dried at 100 °C; and.
− 262 ± 90 μm for fibers dried at 130 °C.

According to Ramesh^48^, the diameter of *in natura* coconut-fiber varies from 10 to 460 μm, and for Castilhos^49^, the diameter of *in natura* coconut-fiber varies from 50 to 400 μm. Galicia-Aldama et al.^30^ report that the average fiber diameter is equal to 15 μm and Gholampour and Ozbakkaloglu^22^ mentioned that the diameter varies from 12 to 25 μm. Thus, the diameters measured using the optical microscope is in accordance with the literature.

According to Canciam et al.^24^, one of the most important physical changes that occurs during drying is the reduction in the external volume of materials.

**Figure 4:**
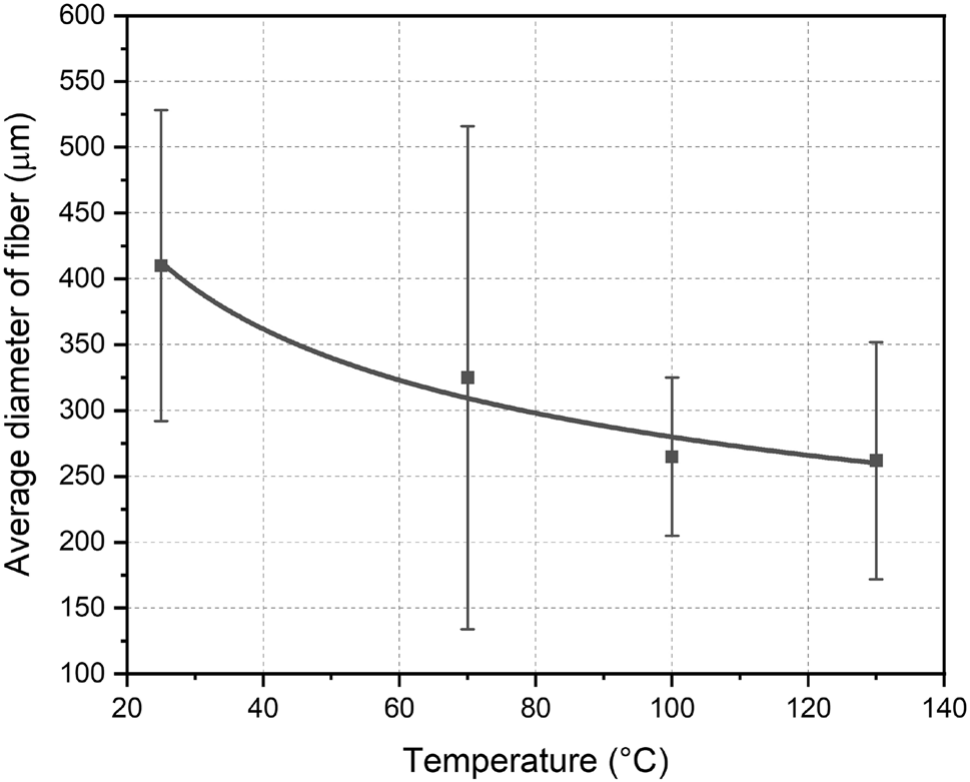
Variations in the average diameter of green coconut-fibers in relation to the drying temperature.

Figure 4 shows the variation in the average coconut-fiber diameter in relation to the drying temperature and a geometric trend line that illustrates this variation. The temperature considered for *in natura* fibers is 25 °C.

It is possible to analyze from the trend line in Fig. 4 that the average diameter of the fibers decreases with drying, which indicates that diametric shrinkage of the fiber occurs due to moisture loss. In addition, it is also possible to verify that from 100 °C onwards there is a small variation in the average diameters and that the greatest variation in diameters occurs for temperatures below 100 °C.

### Thermogravimetric analysis and differential scanning calorimetry

**Figure 5:**
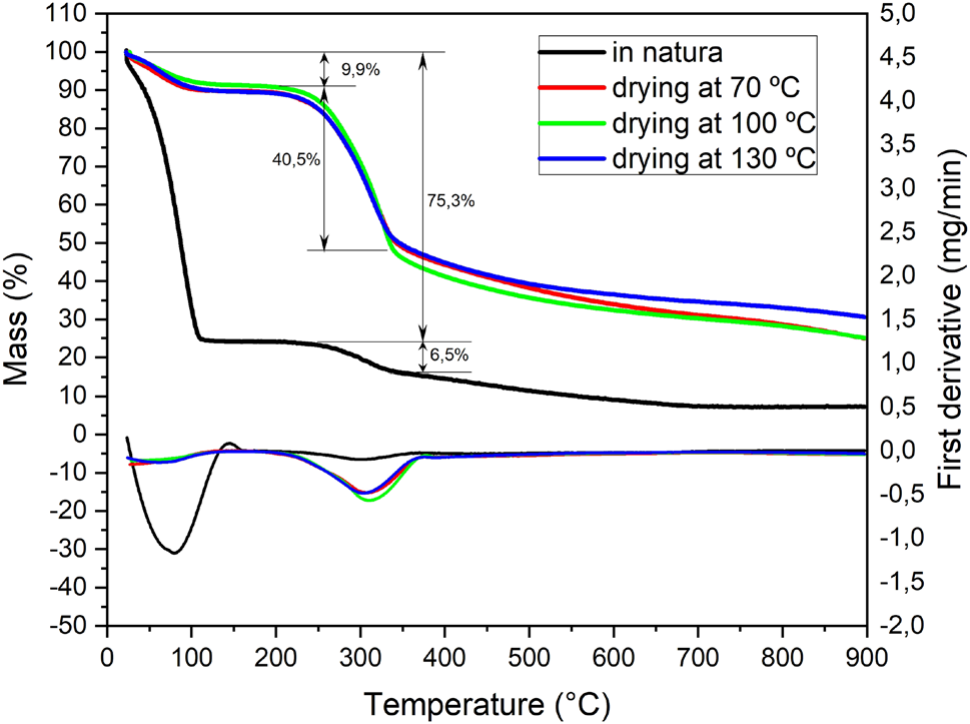
Thermogravimetric curve of coconut-fiber.

Figure 5 presents the mass variation of different coconut-fiber samples with temperature. For the 4 fiber samples analyzed, it is possible to observe 3 well-defined intervals^48^. The first, attributed to the loss of free water, is found between temperatures of 20 and 115 °C. The second interval can be observed between the temperatures of 200 and 375 °C^48^. This range corresponds to the breakdown of cellulose apart from lignin. From 375 °C occurs the last stage, which is related to the degradation of the remaining lignin in the fibers^32^.

In all cases studied, no significant changes were observed in the onset and end temperatures, indicating that drying between 70 and 130 °C did not promote changes in the chemical structure of the fiber components.

As for the results of mass variation, it can be observed that the sample *in natura* presents more than 70% of its mass composed of water. The oven-dried samples showed a similar value of water loss up to 115 °C. The data proves the efficiency of oven drying until mass consistency after fiber extraction^28^.

**Figure 6:**
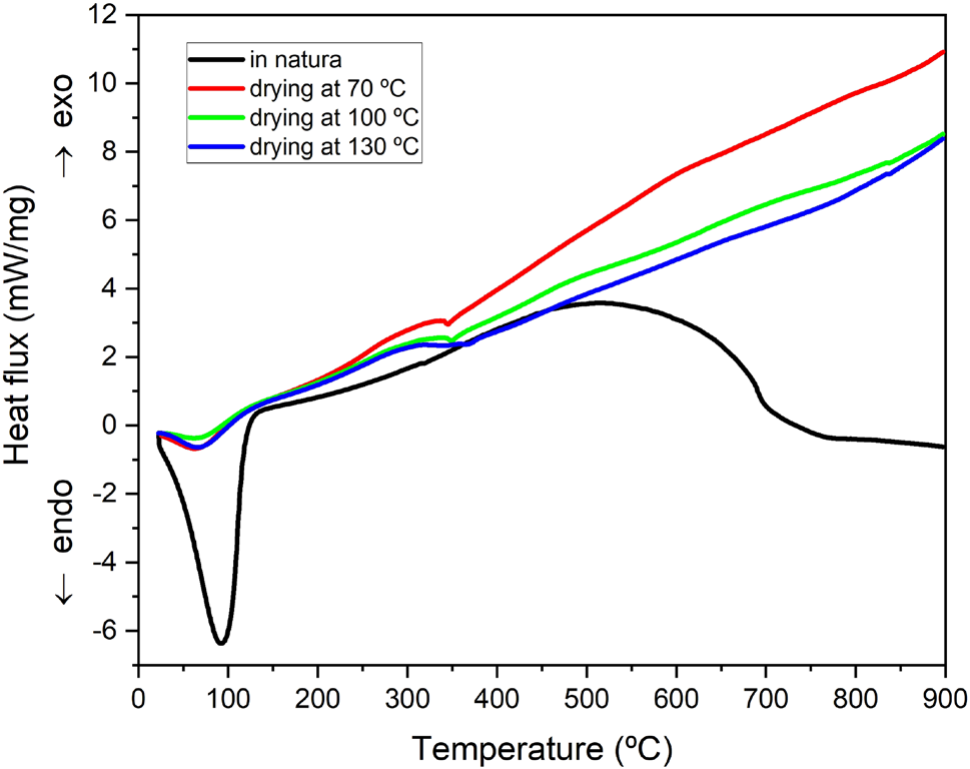
Coconut-fiber DSC curve.

The mass losses can also be observed through the DSC heat flux variation, as shown in Fig. 6. According to the image, peaks with values below 0 are the result of endothermic reactions. Thus, the high peak referring to the *in natura* sample is compatible with the high mass of evaporated water obtained through the analysis of mass variations.

All cases studied show peaks related to water loss. However, some differences were observed. It can be seen that the DSC peak of the *in natura* sample is also slightly shifted to the right compared to the other samples. This difference may be correlated not only to the greater amount of water, but also to the greater difficulty in removing this water from the fiber structures, which is a condition that indicates the existence of water retained in small diameter pore structures^50^.

After the endothermic peaks related to water evaporation, one can observe another thermal phenomenon starting at approximately 350 °C, consisting of a sharp endothermic peak. This peak is related to the depolymerization of hemicellulose and cellulose^50,47^. The other variations especially observed in relation to the others are due to the greater presence of water, modifying the structures of the other components and changing their heat flow pattern^51^.

### Tensile test

**Figure 7:**
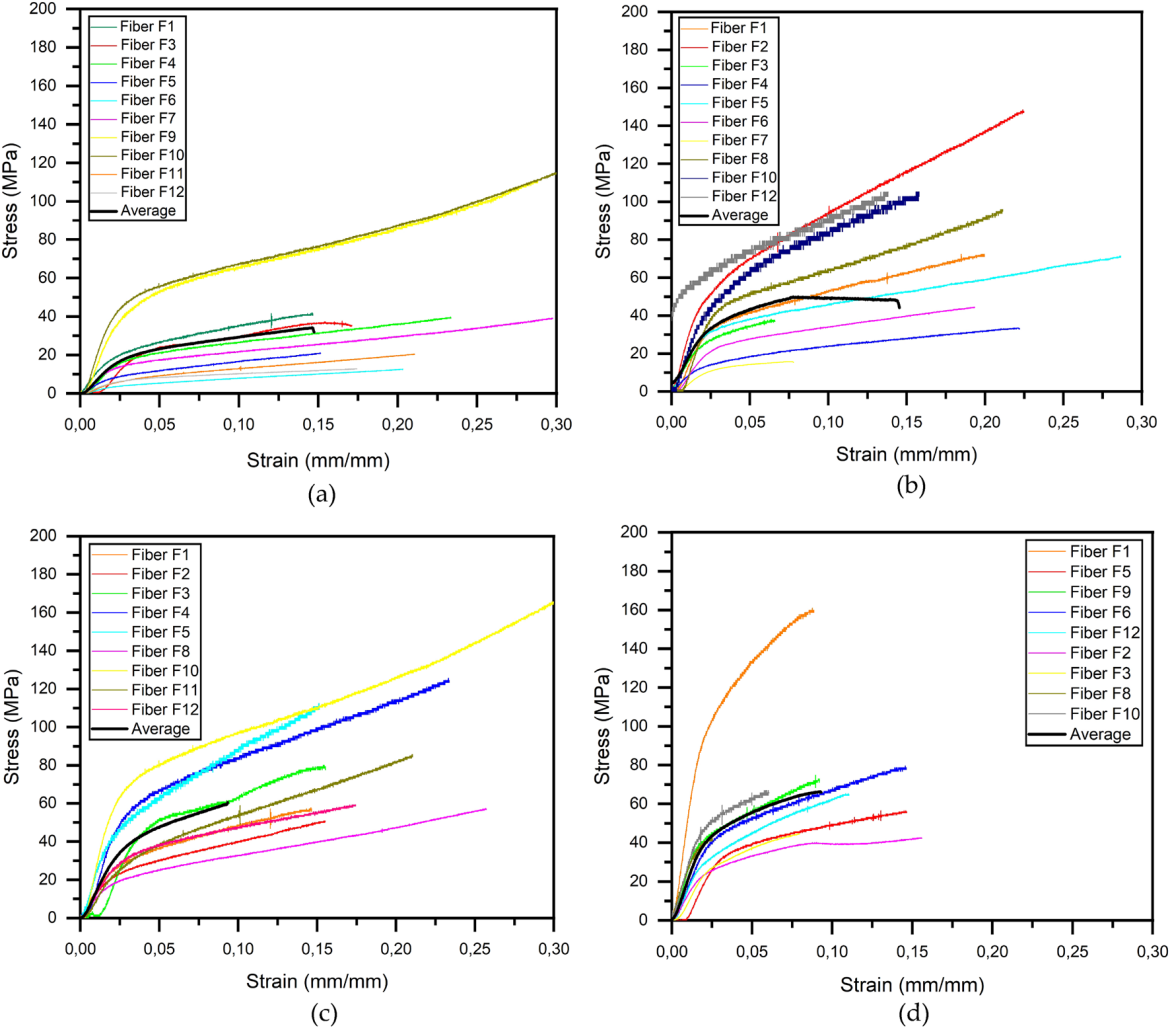
Stress x strain curves of coconut-fibers subjected to tensile testing: (**a**) *in natura*; (**b**) oven dried at 70 °C; (**c**) oven dried at 100 °C and (**d**) oven dried at 130 °C.

The stress vs strain curves for the *in natura* fiber and the fibers subjected to drying are shown in Fig. 7. In Fig. 7a, we have the results of the test for the fiber *in natura*, Fig. 7b results referring to the fibers dried in an oven at 70 °C. In Fig. 7c, the tension and strain values refer to fibers dried in an oven at 100 °C and, finally, in Fig. 7d the values for fibers dried at 130 °C.

All stress vs strain curves showed an initial linear part, followed by a non-linear region and finally a rectilinear ascending region, suggesting some strain hardening. It can also be seen that there is great variability in the results, since the fiber, owing to its vegetable origin, has changes in diameters along the fiber. In addition, depending on the cultivation methods and environmental conditions (soil, water, air and chemicals used), natural fibers can exhibit different behaviors^5^.

The mean experimental stress and strain values for different drying temperatures are listed in Table 3.

**Table 3:** Average stress and strain for the coconut-fibers tested.

Coconut-fibers	Stress (MPa)	Strain (mm/mm)
*In natura*	24.03 ± 8.57	0.073 ± 0.042
Dried at 70 °C	41.6 ± 11.51	0.072 ± 0.042
Dried at 100 °C	40.85 ± 16.45	0.047 ± 0.027
Dried at 130 °C	48.64 ± 17.32	0.047 ± 0.027

For in natura coconut-fibers, there are different values for tensile strength in the literature, as indicated in Table 4.

**Table 4:** Tensile strength for in natura coconut-fiber.

Authors	Stress (MPa)
Ramesh^48^	95–230
Almeida et al.^3^	175
Galicia-Aldama et al.^30^	156–252
Gurunathan et al.^52^	175
Gholampour and Ozbakkaloglu^22^	135–240
Ramamoorthy et al.^37^	131–175
Onuaguluchi and Banthia^25^	175
Malkapuram et al.^38^	131–175

For some fibers tested, tensile strength values were obtained within the values designated by the literature (Table 4), but in terms of average values, the values found for tensile strength were lower, due to the variability of fiber diameter values. There is no uniformity of diameter between fibers and within the same fiber.

According to the ANOVA analysis (Tables 5 and 6), it is observed that the drying temperature was statistically significant in the tensile strength of the fibers (*p* value < 0.05).

**Table 5:** Factor information.

Factor	Levels	Values
Drying temperature	4	0; 70; 100; 130

**Table 6:** Average stress and strain for the coir-fibers tested.

Parameters	Degrees of freedom (d_f_)	Contribution (%)	F value	*P* value
Drying temperature	3	29.68	562.19	0.00

Once the statistical difference was proven, Tukey’s test was performed (Table 7).

**Table 7:** Simultaneous Tukey tests for differences in means.

Difference of levels	Difference of means	T value	*P* value
70–0	17.63	28.29	0.00
100–0	16.83	27.00	0.00
130–0	24.62	39.50	0.00
100–70	− 0.80	− 1.29	0.57
130–70	6.99	11.21	0.00
130–100	7.79	12.50	0.00

It was verified that between the average tensile strengths found considering the drying temperatures of 70 °C and 100 °C there are not statistically differences, for the other temperatures there is a statistical difference.

**Figure 8:**
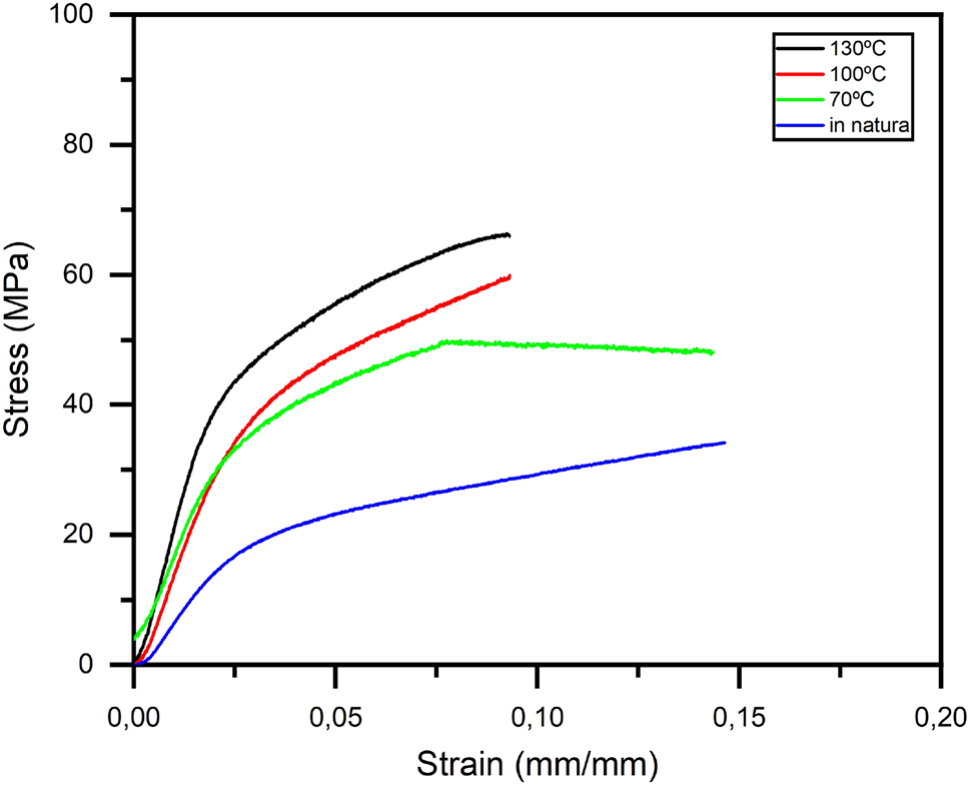
Mean stress vs strain curves of coconut-fibers subjected to different drying temperatures.

However when analyzing the stress vs strain curves of all tested fibers for each drying temperature, Fig. 7, and the average stress vs strain curves for each coconut-fiber drying temperature, Fig. 8, it can be seen that with the increase in drying temperature, the fiber becomes less ductile, presenting brittle behavior and there is a small increase in the longitudinal modulus of elasticity, therefore the coconut-fibers become more rigid.

This increase in modulus of elasticity is higher for temperatures greater than 100 °C. Fibers dried at 70 °C and 100 °C have similar behavior in the elastic phase. Fibers *in natura* support lower tensions but are more ductile because they have a greater deformation before fracture.

**Figure 9:**
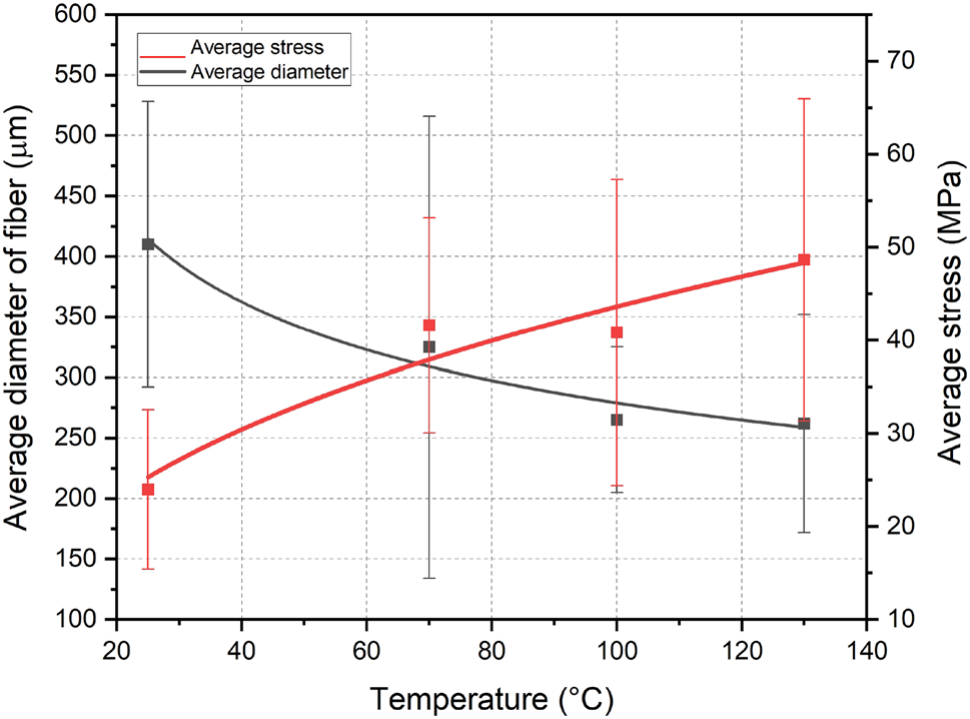
Mean stress and mean diameter curves as a function of different drying temperatures.

Analyzing the average diameter of the fibers with the average tensile stress for *in natura* fibers and for oven-dried fibers, Fig. 9, it can be seen that the diameter decreases as the drying temperatures increase and the opposite occurs with the fiber tensile strength, that is, tensile strength increases with increasing drying temperature.

According to Fuqua et al.^44^ proper drying of the fibers is important for the final mechanical performance of these fibers. Malkapuram et al.^38^ found that there is a decrease in the traction of composites in water-saturated samples compared to dry samples. El Ashcar et al.^53^ also reported that oven-dried cellulose fiber reinforced mortar sample recorded higher flexural strength and lower toughness compared to air-cured or wet-cured samples, making it clear that for effective performance of composites, moisture absorption by Natural plant-based fibers must be controlled.

## Conclusions

Natural fibers are ecological and low-cost materials, widely used, mainly in civil construction, where they can be used in cement matrices, among other applications.

The drying process must be controlled to guarantee the quality of the fibers, especially in relation to mechanical resistance and coloring.

Regarding the influence of the drying temperature of the fibers, it was possible to verify with regard to the chemical analysis of coconut-fibers that there was variation in the results found, as with drying this change in chemical composition is expected. The temperatures employed in the study are lower than the degradation temperature of the constituent elements.

Through scanning electron micrographs, it was possible to conclude that with increasing drying temperature, coconut-fiber underwent surface changes, reducing the diameter of the fiber. By thermogravimetric analysis and differential exploratory calorimetry, it was verified that the in natura coconut-fibers initially suffered a greater mass loss (75.3%), due to the high existing humidity. Dry fibers, on the other hand, showed similar behavior with an initial mass loss of 9.9%.

The different drying temperatures of the coconut-fibers influenced the tensile strength of the fibers, as well as their deformation when subjected to a tensile load.

In the tests carried out, a decrease in the coconut-fiber diameter was verified with the increase in temperature. However, this decrease is not expressive for temperatures above 100 °C. This decrease demonstrates that diametral shrinkage of the fiber occurs due to moisture loss.

Fibers dried in ovens at temperatures above 100 °C, whose drying process was faster, have greater tensile strength when compared to fibers dried in ovens at lower temperatures and *in natura*, but have less ductility, that is, they have less deformation to failure, presenting brittle behavior.

According to the ANOVA analysis of variance, there is not statistically difference between the temperatures of 70 °C and 100 °C, but there are differences between the strains of the fibers subjected to traction.

For the use of the coconut-fiber in cementitious composites, it is advantageous that the fiber has greater ductility. Given the desired ductility, even if the drying process is a little slower, among the three drying temperatures analyzed, the temperature of 70 °C was satisfactory, presenting good tensile strength, combined with good ductility, essential for the application of coconut-fibers in cementitious composites intended for civil construction.

## Data Availability

All data generated or analysed during this study are included in this published article.
